# Toll-Like Receptors and Cancer: MYD88 Mutation and Inflammation

**DOI:** 10.3389/fimmu.2014.00367

**Published:** 2014-07-31

**Authors:** James Q. Wang, Yogesh S. Jeelall, Laura L. Ferguson, Keisuke Horikawa

**Affiliations:** ^1^Department of Immunology, John Curtin School of Medical Research, Australian National University, Canberra, ACT, Australia

**Keywords:** cancer, drug targets, inflammation, lymphoma, MYD88 L265P, pattern recognition receptors, self-nucleic acid, Toll-like receptors

## Abstract

Pattern recognition receptors (PRRs) expressed on immune cells are crucial for the early detection of invading pathogens, in initiating early innate immune response and in orchestrating the adaptive immune response. PRRs are activated by specific pathogen-associated molecular patterns that are present in pathogenic microbes or nucleic acids of viruses or bacteria. However, inappropriate activation of these PRRs, such as the Toll-like receptors (TLRs), due to genetic lesions or chronic inflammation has been demonstrated to be a major cause of many hematological malignancies. Gain-of-function mutations in the TLR adaptor protein MYD88 found in 39% of the activated B cell type of diffuse large B cell lymphomas and almost 100% of Waldenström’s macroglobulinemia further highlight the involvement of TLRs in these malignancies. MYD88 mutations result in the chronic activation of TLR signaling pathways, thus the constitutive activation of the transcription factor NFκB to promote cell survival and proliferation. These recent insights into TLR pathway driven malignancies warrant the need for a better understanding of TLRs in cancers and the development of novel anti-cancer therapies targeting TLRs. This review focuses on TLR function and signaling in normal or inflammatory conditions, and how mutations can hijack the TLR signaling pathways to give rise to cancer. Finally, we discuss how potential therapeutic agents could be used to restore normal responses to TLRs and have long lasting anti-tumor effects.

## Introduction

Pattern recognition receptors (PRRs) are germline-encoded receptors with the ability to relay “danger signals” to the host in order to mediate an early innate immune response. The term “pattern recognition receptors” comes from their ability to recognize specific pathogen-associated molecular patterns (PAMPs) and danger-associated molecular patterns (DAMPs) (1, 2). PRRs can be broadly divided into five distinct subfamilies: Toll-like receptors (TLRs), C-type lectin receptors (CLRs), NOD-like receptors (NLRs), RIG-1-like receptors (RLRs), and AIM2-like receptors (ALRs). These PRR subfamilies differ in their structures, localization patterns, the distinct types of ligands they recognize, and the activation of specific intracellular signaling cascades to mediate a range of responses such as the regulation of gene transcription, cell activation, and proliferation, and the production of pro-inflammatory cytokines, chemokines, and anti-viral molecules (3).

One of the most well characterized PRR is the TLR (4). TLRs are type I transmembrane proteins with an extracellular domain consisting of leucine-rich repeats and a cytoplasmic domain homologous to that of the interleukin (IL)-1 receptor (5, 6). These evolutionarily conserved receptors are absolutely critical for the host innate immune response against many pathogens (7). Activation of TLRs depends on the number of different ligands they may encounter, which is by large, governed by their subcellular localization. Much insight has been gained in recent years on the localization and trafficking of TLRs and the important roles their localization play in the way they recognize their ligands. TLRs can be divided into two groups based on their subcellular localization, either on the cell surface or within intracellular compartments (8). Given the ability of TLRs to recognize a large number of pathogen-associated ligands such as glycoproteins, lipopolysaccharides, flagellin, and viral double-strand or single-strand RNAs or DNAs, TLRs have emerged as an important family of PRRs in shaping both the innate and adaptive immunity (7). However, inappropriate activation of these pathways can often lead to chronic inflammatory diseases or cancer.

This review will focus on TLRs and malignancies associated with the dysregulation of TLR signaling pathways. TLR activation by somatic MYD88 mutations and chronic inflammations has been implicated in a number of hematological malignancies. Targeting the TLR signaling network has gained increasing attention from researchers and clinicians seeking strategies to achieve long lasting anti-tumor outcomes. Here, we discuss the signal transduction and immune regulation by TLRs and the immunological malignancies that manifest from dysregulation of TLR pathways, including how targeting these pathways could be an attractive therapeutic regime.

## Toll-Like Receptors

Toll-like receptors are probably the best studied PRRs that participate in the first line of host defense against pathogens. TLRs belong to an evolutionarily conserved family of adaptors sharing homology with the *Drosophila* protein Toll, which is best known for its essential role in establishing dorsoventral polarity during embryogenesis in insects (9). Amino acid sequencing and hydropathy profiling identified Toll as a type I transmembrane protein with a membrane-spanning segment and multiple tandem leucine-rich repeats directed at the extracellular surface (9). Further biochemical and functional studies conducted on the receptor Toll and its leucine repeats established it as a critical pathogen sensing receptor for recognizing bacteria and fungus in *Drosophila* (10). This study later became critical for the discovery of Toll-like homologs (TLRs) in mammals as mediators of the innate immunity (4, 10, 11).

A total of 10 TLRs have been identified in humans and 12 in mice (7). Due to the small repertoire of TLRs available to recognize a virtually unlimited combination of pathogen-associated patterns, each individual TLR must be able to detect and respond to a large number of pathogens ranging from bacteria, fungi, protozoa, and viruses (12, 13). For instance, TLRs 1, 2, and 6 recognize lipo-, glycol-, and acyl-peptides expressed on the surfaces of many Gram-positive and Gram-negative bacteria and mycobacteria (7). Additional cooperation between TLRs 1, 2, and 6 enables them to further discriminate different microbial components (14). TLR4 recognizes lipopolysaccharide components of the cell wall of Gram-negative bacteria through its co-receptor MD-2 (15, 16). In addition, TLR4 can also recognize endogenous ligands such as heat-shock proteins, extracellular matrix components including fibronectin, hyaluronic acid, and heparin sulfate in response to tissue injury (7). The nucleic acid sensing subfamily of TLRs consists of TLRs 3, 7, and 9 and exhibit unique endosomal localization in contrast to the surface expression of the other TLRs (17). These TLRs have the ability to detect nuclear material such as ssRNAs, dsRNAs, and dsDNAs and are vital for anti-viral responses (18–21). Importantly, these nucleic acid sensing TLRs must discriminate between foreign and self-nuclear material to prevent autoimmunity. Due to the relative lack of specificity of TLRs compared to the B cell receptors (BCRs), restriction of self-TLR activation must be achieved through other means. TLRs are protected from engaging self-nuclear material by Unc93b mediated restriction to the endosome (22). In such way, self-nucleic acids are prevented from entering the endosome, but foreign material can enter via endocytosis and be processed in the acidified endosomes in order to activate the endosomal TLRs (23, 24).

**Figure 1:**
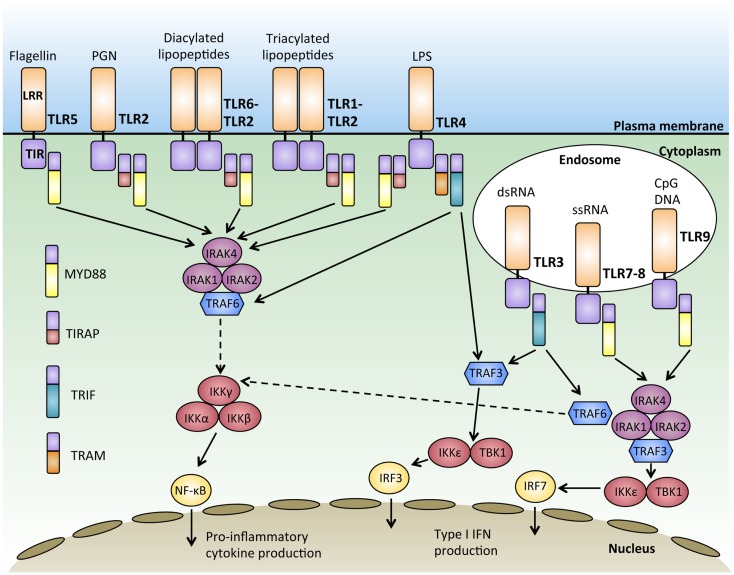
**Signal transduction downstream of MYD88-dependent and independent pathways**. Activation of Toll-like receptors (TLRs) through binding of their ligand leads to receptor dimerization and the recruitment of adaptor proteins such as MYD88, TIRAP, TRIF, and TRAM. Most of the TLRs form homodimers upon activation while TLR2 can also form heterodimers with either TLR6 or TLR1 to recognize diacylated and triacylated lipopeptides, respectively. Downstream signals are propagated through the activation of IRAKs-TRAF6 and the IKK complex, culminating in the activation of transcription factors such as nuclear factor-κB (NFκB) and interferon-regulatory factors (IRFs), which regulate the production of pro-inflammatory cytokines and type 1 interferon (IFNs).

Together, the 10 human TLRs can recognize a virtually unlimited combination of pathogens, however, the downstream signaling pathways they share are striking. All TLRs except for TLR3 signal through the adaptor protein MYD88 (25). Upon ligand binding, TLRs induce the dimerization of their ectodomains, bringing the cytoplasmic TIR domains together, and initiating a signaling cascade via signal adaptor molecules. The four main TLR adaptor molecules are the myeloid differentiation response protein 88 (MYD88), Toll-interleukin 1 receptor (TIR) domain containing adaptor protein (TIRAP; also known as MAL), TIRAP inducing IFN-β (TRIF), and TRIF-related adaptor molecule (TRAM) (Figure 1). These adaptors are used in various combinations by the different TLRs, but these signaling pathways can be broadly classified into either MYD88 dependent or MYD88 independent.

## MYD88 Dependent TLR Signaling

With the exception of TLR3, all TLRs initiate a MYD88-dependent signaling pathway (26). The signal adaptor protein MYD88 contains two main conserved protein domains; a C-terminal TIR and a N-terminal death domain (DD) (27, 28). Upon TLR activation, MYD88 is recruited to the TIR domain of the activated TLR via TIR–TIR interaction (29). The serine–threonine kinase, IL1-receptor associated kinase 4 (IRAK4), is then recruited to MYD88 through the interaction of their DD domains. IRAK4 then recruits and phosphorylates IRAK1 and IRAK2 to form a structure known as the “Myddosome” (30). Phosphorylation of IRAKs 1 and 2 allows them to interact with the E3 ubiquitin ligase, TRAF6, via their TRAF binding domain (31). TRAF6 then ubiquitylates and activates TAK1 (32), which has the dual ability to activate both the NFκB pathway and the mitogen-activated protein kinase (MAPK) pathway (26). In resting cells, NFκB dimers are sequestered in an inactive form in the cytoplasm by the IκB protein (33). During NFκB activation, TAK1 phosphorylates and activates IκB kinase β (IKKβ), which in turn phosphorylates IκB, targeting it for proteosomal degradation (34). The degradation of IκB releases NFκB, enabling it to enter the nucleus and bind to sequences known as κB sites to activate transcription of genes (35). TAK1 also activates the MAPK pathway, leading to the activation of c-Jun N-terminal kinase (JNK), which activates the Jun family of transcription factors (36) (Figure 1).

The MYD88-dependent pathway can be initiated by TLR5 and TLR7-9 using the adaptor MYD88 alone, while the adaptor protein TIRAP is required with MYD88 to initiate signaling downstream of TLR2 and TLR4 (37, 38). In this subset of TLRs, TIRAP acts as a sorting molecule that is necessary for efficient recruitment of MYD88 to the activated TLRs to initiate signal transduction to activate NFκB and produce pro-inflammatory cytokines (39). During TLR 7 and 9 activation, MYD88 also recruits TRAF3 to activate TBK1 and IKKε, which phosphorylates the transcription factor interferon-regulatory factor 7 (IRF7) and leads to IFN-α production (40, 41). IFN-α production, as with production of other IFNs, is particularly important for anti-viral responses (42) (Figure 1).

## MYD88 Independent TLR Signaling

MYD88-independent TLR3 signaling requires the adaptor molecule TRIF to activate downstream signaling pathways, including the activation of IRF3 and the production of IFN-β (43). TRIF has also been known to participate in signaling downstream of TLR4 for type 1 interferon responses (44). Upon ligand binding, TRIF recruits TRAF3, which acts as a scaffold for the activation of the IKKs, TBK1, and IKKε, leading to the phosphorylation and activation of the transcription factor IRF3 and IFN-β transcription (45, 46). While TLR3 can activate this pathway using TRIF alone, the adaptor TRAM is required for TLR4, where TRAM facilitates the recruitment of TRIF to TLR4 (47). Upon the activation of TRIF, TRAF6 is recruited, which then activates TAK1 through ubiquitination and leading to the subsequent activation of NFκB (48)(Figure 1). Interestingly, TRAF3 has been shown to play important roles in regulating both the MYD88 dependent and independent response through its differential ubiquitination (49). MYD88-independent signaling triggers the non-degradative self-ubiquitination of TRAF3, promoting IRF3 activation. On the other hand, the MYD88-dependent pathway results in the degradative ubiquitination of TRAF3 and the activation of TAK1 (49). In this manner, TRAF3 acts to balance pro-inflammatory and IFN response by the MYD88 dependent and independent pathways.

## Hematological Malignancy and MYD88 Mutation

**Figure 2:**
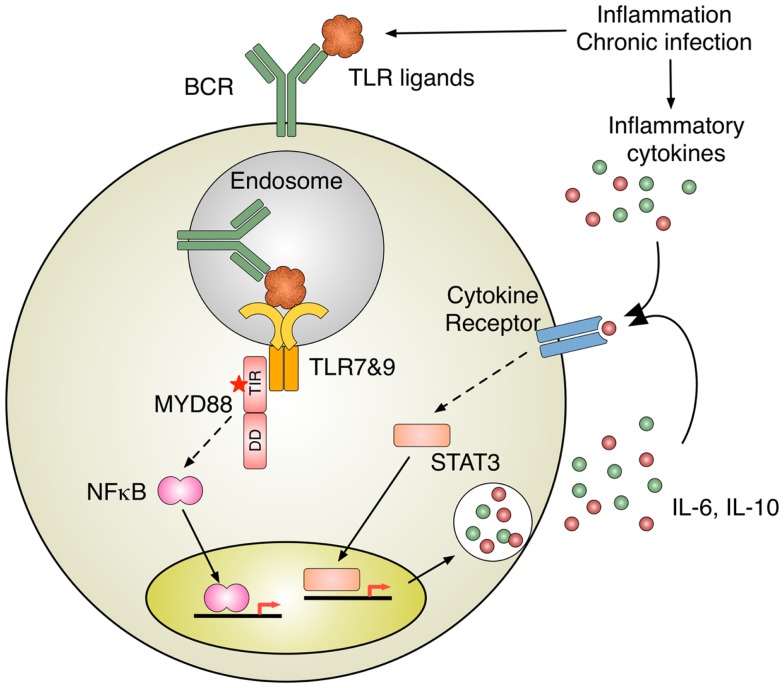
**Inflammation induces B cell activation**. TLR activation by nucleic acid–protein complexes derived from inflammation and MYD88 mutation B cell antigen receptor (BCR) delivers nucleic acids–protein complexes to TLR-containing endosomes, where MYD88 initiates the activation of intracellular signaling pathways, such as NFκB. Intriguingly, oncogenic MYD88 mutations require intact TLR apparatuses to recognize nucleic acids. One of the potential sources for TLR ligands is nucleic acid–protein complexes derived from inflammation and chronic infection. A subset of ABC-DLBCL shows constitutively activation of JAK-STAT3 pathway, presumably due to autocrine stimulation by IL-6 and IL-10. The activation of cytokine receptor signaling can also be induced by inflammatory cytokines in the milieu of inflammation and chronic infection.

Inappropriate activation of TLRs due to the somatic acquisition of gain-of-function mutations in the TLR adaptor protein MYD88 has been implicated in many hematological malignancies. Activated B cell type diffuse large B cell lymphoma (ABC-DLBCL), a particularly aggressive subtype of DLBCL whose pathogenesis relies on constitutively active NFκB, frequently accumulates MYD88 mutations. 39% of tumor samples contain mutations in MYD88, and strikingly, 29% of those mutations result in a single nucleotide change from leucine into proline at position 265 (L265P) (50). shRNA knockdown of MYD88 in lymphoma cell lines demonstrated that MYD88 mutations are critical for their survival and high NFκB transcription factor activity (50). A hyperphosphorylated isoform of IRAK1 was strongly associated with the L265P mutant form of MYD88, suggesting that this mutation is a gain-of-function mutation that leads to the constitutive activation of downstream IRAKs (50). The effects of the L265P mutation include increased NFκB activity as well as increased JAK-STAT3 signaling and the production of pro-inflammatory cytokines such as IL6, IL10, and IFN-β (50). The production of these cytokines further activates JAK-STAT3 signaling as part of an autocrine loop that enhances the survival of the lymphoma cells (51, 52) (Figure 2).

MYD88 mutations have since emerged in a number of other human malignancies, with the L265P mutation found in including almost 100% of Waldenström’s macroglobulinemia (WM), 2–10% of chronic lymphocytic leukemia (CLL), 69% of cutaneous diffuse large B cell lymphoma (CBCL), and 38% of primary central nervous system lymphoma (PCNSL) (previously reviewed in Ref. (53)). However, the effect of single MYD88 L265P mutation on tumor growth is confounded by the accumulation of other potential damaging mutations in the same malignant clones. Recently, a retroviral gene transfer strategy to study the effects of single MYD88 mutation in other wise normal mature B cells found that the MYD88 L265P mutation alone was able to drive limited rounds of mitogen independent B cell proliferation both *in vitro* and *in vivo* (54). Nevertheless, the drive for B cell proliferation was dependent on intact nucleic acid sensing TLR activity since *Unc93b1^3d^* mutation or *Tlr9* deficiency inhibited the proliferation of MYD88 L265P B cells *in vitro* (54). Other studies have also shown that oncogenic MYD88 depends on TLRs by using the depletion of UNC91B1, PRAT4A, and CD14 in ABC-DLBCL lines as well as by using pharmacological inhibitors to TLR7 and TLR9 (55). Given that intact TLR activity is critical for lymphoma cells carrying MYD88 mutations, targeting this pathway appears to be attractive for treating these malignancies. Indeed, blocking endosome acidification using chloroquine selectively inhibits MYD88 L265P mutation driven B cell proliferation *in vitro* (54). The use of chloroquine to treat hematological malignancies should be further explored, as evidence suggests that there is a strong involvement of the activation of nucleic acid sensing TLRs that depends on normal endosome acidification in promoting proliferative abnormality in these tumors.

## Hematological Malignancy and Inflammation

Remarkably, inflammation enables most of the key cellular and molecular capabilities that are required for carcinogenesis, such as genomic instability, proliferative abnormality, and reprograming of the stromal environment (56). Although, the mechanisms by which inflammation promotes neoplastic transformation are not fully understood, it is apparent that, in many cases, tumor development is linked to chronic inflammation (57, 58).

The link between inflammation and tumor formation was first speculated by Virchow in the 1800s as he observed that tissue injury and inflammation induced by irritants could promote cell proliferation (59). Infection has been accepted as a major driver of inflammation-induced tumorigenesis, with up to one-fifth of all cases of cancer associated with infection (60, 61). For instance, persistent *Helicobacter pylori* infection is associated with gastric cancer and mucosa-associated lymphoid tissue (MALT) lymphoma, infections with hepatitis B and C viruses are associated with hepatocellular carcinoma, and infections with *Bacteroides* species are linked to colon cancer (62, 63). The inflammatory response triggered by infection is a part of normal host defense to eliminate the pathogen. However, some tumorigenic pathogens subvert host immunity and establish persisting infections, leading to chronic inflammation (64, 65).

**Figure 3:**
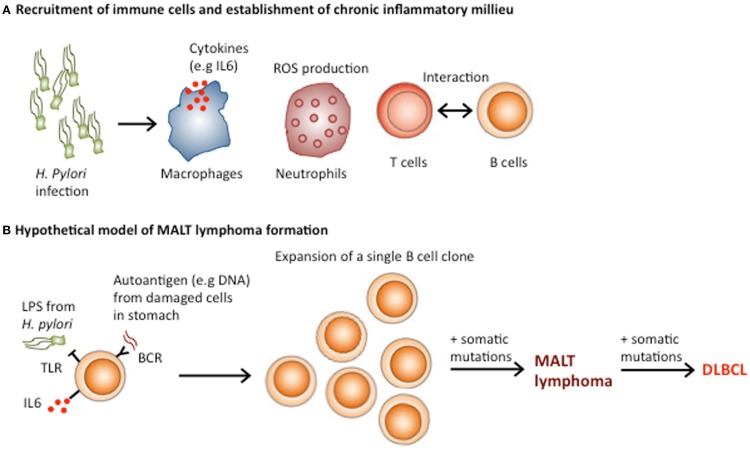
**Role of *Helicobacter pylori* in the pathogenesis of gastric MALT lymphoma**. **(A)** *H. pylori* infection results in buffering of the gastric pH, which allows immune cell infiltration and the establishment of MALT. The presentation of *H. pylori* antigens by dendritic cells recruits and activates T cell responses, which enhance B cell activation through CD40–CD40L interactions. **(B)** MALT lymphoma may result from the transformation of a single B cell clone, which initially formed part of the polyclonal B lymphocyte response against *H. Pylori*. Both direct activation of TLR signaling by *H. pylori* and chronic BCR signaling from engagement of autoantigens from damaged stomach cells and the B cell receptor, in addition to T cell-B cell co-stimulation could be involved in the expansion of the single neoplastic B cell clone. Acquisition of additional genomic lesions could transform MALT lymphomas into more aggressive DLBCL.

Persistent inflammation establishes a microenvironment, which contains macrophages, dendritic cells, natural killer cells, and T and B lymphocytes in addition to the surrounding stroma (66) (Figure 3A). These diverse cells communicate with each other by means of direct contact or cytokine and chemokines, which influence tumor formation and growth (67, 68). This network of inflammatory cells promotes the formation of cancerous cells, which further complicates the initial chronic inflammation induced by infection. The neoplastic cells trigger anti-tumor immunity, which further adds to the established inflammation. Early during tumor formation, whether tumor-promoting inflammation or anti-tumor immunity follows seems to be stochastic and is influenced by a combination of cell-intrinsic and cell-extrinsic processes (69, 70). In established cancers, pro-tumor inflammation seems to be favored, as without therapeutic intervention advanced tumors rarely regress.

Continuous stimulation of TLRs by microbial products constitutively engages the activation of the NFκB and STAT3 transcription factors, which exert pro-cancerous activity through multiple effectors (62, 71, 72). Additionally, the production of cytokines by the host inflammatory cells activates these transcription factors (62) (Figure 2). These cytokines facilitate the establishment of feed-forward signal amplification loops, which ultimately promote cell proliferation and resistance to cell death. For instance, the expression of the anti-apoptotic proteins Bcl-2 and Bcl-X_L_ is promoted by both NFκB and STAT3, as is the expression of c-IAP1, c-IAP2, Mcl-1, c-FLIP, and survivin (62, 72). Moreover, both transcription factors interfere with p53 expression and function, representing another potential tumor-promoting mechanism (73).

An additional mechanism linking inflammation to tumor formation is the expression of activation-induced cytidine deaminase (AID), an enzyme that promotes immunoglobulin gene class switching by catalyzing deamination of cytosines in DNA (74). In addition to B lymphocytes, where it was originally discovered, AID is overexpressed in many cancers of diverse origin, and its expression is induced by inflammatory cytokines in a NFκB-dependent manner (74). AID induces genomic instability and increases mutation probability during the error-prone joining of double-stranded DNA breaks. This mutagenic process causes mutations in critical cancer-associated genes such as Tp53 and c-Myc (75, 76).

## *Helicobacter pylori*, Inflammation and MALT Lymphoma

MALT lymphomas, which occur in the context of chronic inflammation caused by infectious agents, such as *H. pylori* (gastric lymphoma), *Chlamydia psittacii* (ocular adnexal lymphoma), and *Borrelia burgdorferi* (cutaneous lymphoma) are a prime example of lymphoid malignancies associated with chronic inflammation (77, 78). Interestingly, in some patients, gastric MALT lymphoma and diffuse large B cell lymphoma (DLBCL) co-occur, indicating that MALT lymphomas can develop into more aggressive DLBCL (79). The pathogenesis of MALT lymphoma involves several steps, which result in the transformation of a single B cell clone, initially part of the polyclonal B lymphocyte response against *H. Pylori* into a monoclonal tumor (78) (Figure 3B). Under physiological conditions, the stomach lacks MALT because the low pH prevents the survival of lymphocytes in the gastric wall. However, *H. pylori* infection results in buffering of the gastric pH owing to the secretion of bacterial urease. The decreased acidity of stomach environment, along with the presence of the infection, triggers lymphoid infiltration and the establishment of MALT (78).

Subsequently, the continuous presence of *H. pylori* induces an upregulation of TLR4 and MD-2 expression in gastric epithelial cells, which contributes to establishing a persistent inflammatory environment (80–82). Although, the role of TLRs in the pathogenesis of MALT lymphoma has been poorly investigated, the immune response to chronic stimulation by *H. pylori* infection is thought to induce NFκB activation in B cells, which plays a crucial part in the development of MALT lymphoma (83, 84). In addition, the presentation of *H. pylori* by dendritic cells recruits and activates T cell responses, which enhance B cell activation through CD40–CD40L interactions (85) (Figure 3A). Thus, both direct activation of TLR signaling by *H. pylori* and T cell-mediated B cell activation could be involved in the pathogenesis of MALT lymphoma (86).

Interestingly, several lines of evidence indicate that chronic antigen stimulation precedes MALT lymphoma pathogenesis. The rearranged *IGVH* genes from MALT lymphomas have a high frequency of variants, which have been implicated in autoantibody production (87). In addition, approximately half of the MALT lymphoma cases display evidence of intraclonal variation in the *IGVH* locus, indicating that continued antigenic stimulation is a key driver of clonal B cell expansion (87). As both somatic hypermutation and intraclonal variations are antigen-driven processes, their occurrence in gastric MALT lymphoma strongly indicates a role for antigens during both initiation and progression of this neoplasm.

Remarkably, tumor-derived immunoglobulins from MALT lymphomas bind to various autoantigens as well as *H. pylori* with varying affinities (87). The autoantigens include DNA and stomach-associated antigens, which could be abundant in the MALT-microenvironment under a situation of continuous inflammation. Given that *H. pylori* eradication with antibiotics is the preferred therapy for patients with *H. pylori*-positive gastric MALT lymphoma (88, 89), and the evidence that MALT lymphoma cells proliferate when stimulated with *H. pylori* in tissue culture, one possible hypothesis is that neoplastic B cells receive proliferative signals from both the B cell receptor and TLRs, which are continuously and simultaneously engaged by self-antigens and LPS from *H. pylori* respectively. Thus, the eradication of *H. pylori* by antibiotics disrupts a critical ‘weak’ link in the inflammatory process, which gradually resolves and shuts off the supply of autoantigens available to lymphoma cells.

## Role of Inflammation and Cytokines in CLL and Multiple Myeloma

It is apparent that antigenic stimulation, autoimmunity, and inflammation contribute to the development of CLL (90). One mechanism through which these stimuli promote CLL development is induction of B cell activating factor (BAFF), a member of the TNF family, recently shown to accelerate development of CLL-like disease in mice (91). In addition, cytokines such as IL6 and interactions with bone marrow stromal cells support CLL expansion and suppress apoptosis through the expression of Bcl-2, Survivin, and Mcl-1 (92, 93). Increased IL6 production activates the JAK-STAT, MAPK, and PI3K pathways to promote cell survival, proliferation, and resistance to apoptosis (94–96), with the constitutive activation of STAT3 being a hallmark for CLLs (97, 98). Similarly, through the secretion of IL6, TNF-α, and BAFF, bone marrow stromal cells promote the survival of neoplastic plasma cells and also confer drug resistance in multiple myeloma (99). Interestingly, IL6-deficient mice are resistant to induction of multiple myeloma (100, 101). Thus, despite cell-intrinsic constitutive NFκB activation, multiple myeloma cells depend on an extrinsic source of IL6 for their development and survival. High levels of plasma IL6 have been associated with increased disease progression and decreased survival, thus providing the rationale for the evaluation of combination therapies including drugs targeting IL6 for the treatment of this malignancy (102–104).

## Targeting Inflammation and TLRs in Cancer

Constitutively, active NFκB signaling due to the aberrant activation of TLRs during chronic inflammation or by MYD88 mutation determines the poor clinical outcome of many hematological malignancies. Desirable outcomes in treating these diseases can be achieved by using a combination of inhibition of signal transducers and transcription factors, sequestration of chemokines and cytokines that sustain inflammatory cells, and the depletion of immune or inflammatory cells that promote tumor development.

Gain-of-function MYD88 mutations have emerged as a potent driver of constitutively active NFκB signaling in many tumors. Targeting this pathway is likely going to be useful as part of a multi-component therapy for many hematological malignancies that are addicted to NFκB activity for their survival. MYD88 signaling is critically dependent on its homo-dimerization through conserved residues within the BB-loop structure of the TIR domain (29, 105). Interfering with this interaction by heptapeptides mimicking the BB-loop has achieved significant reduction in NFκB activity (106). Another novel synthetic compound, ST2825, developed by the same group of researchers is currently under pre-clinical evaluation for the treatment of chronic inflammatory diseases (107). Other peptide-based synthetic small molecule inhibitors such as hydrocinnamoyl-l-valyl pyrrolidine (compound 4a) and Pephinh-MYD88 have also been developed to target MYD88 dimerization in the treatment of lymphoma patients with MYD88 mutations (108). However, these potential MYD88 specific therapeutic options are yet to be trialled in large clinical cohorts.

Constitutive NFκB activity in certain lymphoid tumors suggests that the activation of this pathway is crucial for their survival and thus making them attractive drug targets for anti-cancer therapy (62, 72, 109, 110). However in most cases, such therapy is likely to be effective only in combination with more conventional approaches. Furthermore, as genotoxic therapies often lead to NFκB activation in remaining malignant cells, it makes sense to combine genotoxic dugs with NFκB inhibitors to overcome drug resistance. However, prolonged NFκB inhibition can result in severe immune deficiency and may lead to neutrophilia and greatly enhanced acute inflammation due to enhanced IL1β secretion. Such complications as well as increase propensity for liver damage have hindered the clinical development of NFκB and IKKβ inhibitors (57, 111, 112). An attractive alternative target is the STAT3 transcription factor and the signaling pathway that leads to its activation (113, 114). Several STAT3 and JAK2 inhibitors have been described and shown to inhibit the growth of various cancers that exhibit STAT3 activation (115, 116). So far, none of the complications associated with NFκB inhibitor have been reported for STAT3 or JAK2 inhibitors.

It is unlikely that inhibition of NFκB or STAT signaling alone will be sufficient for tumor regression, yet the combination of an NFκB inhibitor and an apoptosis inducing drug or cytokine could be highly effective. Selective inhibition of NFκB in cancer cells blocks the stimulatory effect of TNF and markedly increases susceptibility to TRAIL-induced cell death, resulting in tumor regression (117, 118). NFκB inhibition and anti-TNF therapy, together with the administration of IFN or TRAIL might offer an attractive combined strategy for immunomodulatory cancer therapy. A recent study has found such synergy between lenalidomide and the BTK inhibitor Ibrutinib in killing ABC-DLBCL by the induction of IRF7 and IFN-β production to cause cell cycle arrest and apoptosis (119, 120). Combinatorial strategies provide a distinct advantage where by certain IFN induced side-effects might be diminished after NFκB inhibitor treatment, shifting the balance of cytokines in the tumor microenvironment to promote tumor regression.

Although it is widely accepted that dampening inflammation and diminishing TLR activity are beneficial for tumor regression, several new lines of evidence have emerged to suggest that TLR agonists could be used as potent anti-tumor agents. When treated with a TLR9 agonist, type B CpG oligodeoxynucleotides (CpG-B ODNs), and CLL B cells that selectively express high levels of TLR9 undergo profound apoptosis by the activation of STAT1, reduction of Bcl-xl pro-survival protein, and elevation of Fas and Fas ligand (121). TLR9 triggered apoptosis seems to be dependent on the altered NFκB status of lymphoma cells compared to normal cells. Moreover, the use of TLR agonists has been known to activate the cognate immune system against cancer cells (122–124). TLRs in lymphoid malignancies appear to be a “double-edged sword” in actively driving disease progression in some but exhibit tumor regressive roles in others. Activation of TLRs by MYD88 mutations has often been associated with poor clinical outcome in lymphoma patients. However, a recent study has reported improved patient survival in a subset of young CLL patients with the identical mutation (125). Interestingly, patients with MYD88 mutations were much younger and had lower expression of CD38 and ZAP-70 than patients with unmutated MYD88. CD38 expression on CLL cells is important for their proliferation and chemotaxis through a signaling pathway involving ZAP-70 (90). Elevated CD38 expression often marks CLL patients with poor clinical outcome and responsiveness to therapy (90). Complex interactions between MYD88 mutation, IGHV mutation status, and CD38 and ZAP-70 levels confound the explanation behind why patients with MYD88 mutations had reduced CD38 expression and show better survival (125).

## Conclusion

Pattern recognition receptors protect us from danger and damage associated signals, however, inappropriate activation of these pathways can cause cancer. TLRs can also use ubiquitously available self-ligands such as our own DNA to drive aberrant cell growth when the adaptor protein MYD88 is mutated. This recent finding is one of the many pieces of supportive evidence for Virchow’s hypothesis that chronic inflammation is linked with cancer development. Studies into mutations in the TLR signaling pathways have significantly advanced our understanding on the involvement of TLRs in cancer. However, the potential for targeting TLRs as anti-cancer therapy remains an area that is not yet fully understood. Often TLRs act as a “double-edged sword” in cancer, over active TLR signal provides a microenvironment that is necessary for malignant cell proliferation; on the other hand, TLR agonists can also be used to inhibit cancer cell growth. A better understanding of the involvement of TLRs in cancer would help in tipping the balance between tumor stimulatory and inhibitory effects and the development of novel anti-cancer agents.

## Conflict of Interest Statement

The authors declare that the research was conducted in the absence of any commercial or financial relationships that could be construed as a potential conflict of interest.
